# Extracellular and Mixotrophic Symbiosis in the Whale-Fall Mussel *Adipicola pacifica*: A Trend in Evolution from Extra- to Intracellular Symbiosis

**DOI:** 10.1371/journal.pone.0011808

**Published:** 2010-07-27

**Authors:** Yoshihiro Fujiwara, Masaru Kawato, Chikayo Noda, Gin Kinoshita, Toshiro Yamanaka, Yuko Fujita, Katsuyuki Uematsu, Jun-Ichi Miyazaki

**Affiliations:** 1 Chemo-Ecosystem Evolution Research (ChEER) Team, Japan Agency for Marine-Earth Science and Technology, Yokosuka, Japan; 2 Keikyu Aburatsubo Marine Park, Miura, Japan; 3 Graduate School of Biosphere Science, Hiroshima University, Higashi-Hiroshima, Japan; 4 Graduate School of Natural Science and Technology, Okayama University, Okayama, Japan; 5 Graduate School of Life and Environmental Sciences, University of Tsukuba, Tsukuba, Japan; 6 Department of Technical Services, Marine Work Japan Ltd., Yokosuka, Japan; 7 Faculty of Education and Human Sciences, University of Yamanashi, Kofu, Japan; University of Canterbury, New Zealand

## Abstract

**Background:**

Deep-sea mussels harboring chemoautotrophic symbionts from hydrothermal vents and seeps are assumed to have evolved from shallow-water asymbiotic relatives by way of biogenic reducing environments such as sunken wood and whale falls. Such symbiotic associations have been well characterized in mussels collected from vents, seeps and sunken wood but in only a few from whale falls.

**Methodology/Principal Finding:**

Here we report symbioses in the gill tissues of two mussels, *Adipicola crypta* and *Adipicola pacifica*, collected from whale-falls on the continental shelf in the northwestern Pacific. The molecular, morphological and stable isotopic characteristics of bacterial symbionts were analyzed. A single phylotype of thioautotrophic bacteria was found in *A. crypta* gill tissue and two distinct phylotypes of bacteria (referred to as Symbiont A and Symbiont C) in *A. pacifica*. Symbiont A and the *A. crypta* symbiont were affiliated with thioautotrophic symbionts of bathymodiolin mussels from deep-sea reducing environments, while Symbiont C was closely related to free-living heterotrophic bacteria. The symbionts in *A. crypta* were intracellular within epithelial cells of the apical region of the gills and were extracellular in *A. pacifica*. No spatial partitioning was observed between the two phylotypes in *A. pacifica* in fluorescence *in situ* hybridization experiments. Stable isotopic analyses of carbon and sulfur indicated the chemoautotrophic nature of *A. crypta* and mixotrophic nature of *A. pacifica*. Molecular phylogenetic analyses of the host mussels showed that *A. crypta* constituted a monophyletic clade with other intracellular symbiotic (endosymbiotic) mussels and that *A. pacifica* was the sister group of all endosymbiotic mussels.

**Conclusions/Significance:**

These results strongly suggest that the symbiosis in *A. pacifica* is at an earlier stage in evolution than other endosymbiotic mussels. Whale falls and other modern biogenic reducing environments may act as refugia for primal chemoautotrophic symbioses between eukaryotes and prokaryotes since the extinction of ancient large marine vertebrates.

## Introduction

Deep-sea bathymodiolin mussels (Bivalvia: Mytilidae) thrive in reducing environments such as hydrothermal vents, hydrocarbon seeps, whale falls and sunken wood and have chemoautotrophic and/or methanotrophic symbiotic relationships with proteobacteria [1]–[13]. These mussels rely primarily on their symbionts for nutrition, although some may also be facultative filter feeders [14]–[17].

Symbiont-harboring deep-sea mussels (primarily subfamily Bathymodiolinae) are hypothesized to have derived from asymbiotic shallow-water relatives by way of sunken wood falls, which were proposed to act as evolutionary stepping-stones for the introduction of chemoautotrophy-dependent invertebrates into vent and seep environments based on the results of molecular phylogenetic analyses of mytilid mussels [18]. Further phylogenetic analyses of sunken-wood mussels strongly supported the “wooden steps to deep-sea vent” hypothesis [12], [19].

In contrast, the fossil records of mytilid bivalves at seeps from the Jurassic (150 Ma) and the oldest occurrence of *Bathymodiolus* species at seep sites from the upper middle Eocene (between 37 and 47 Ma) are earlier than the evolution of the larger whales [20]. It was suggested that whale-fall taxa were derived from seep relatives because 76% of all seep mollusks originated before the major radiation of unequivocal ocean-going whales in the mid-Oligocene [21]. As such, whale falls may have presented new niches for taxa that were already adapted to ephemeral reducing environments rather than an evolutionary stepping-stone toward vents and seeps [22]. The evolution of symbiont-harboring mussels is unclear because molecular and fossil results are inconsistent.

Integrated intracellular symbiosis probably originated from an extracellular association between organisms [23], [24]. All bathymodiolin mussels reported to date from hydrothermal vents and seeps exhibit intracellular symbioses, although the symbiotic form of a new bathymodiolin mussel collected from the Juan de Fuca hydrothermal vents was uncertain because of the poor condition of fixed tissue [1], [5], [7], [25], [26]. Meanwhile, four unidentified mytilids collected from wood falls in the west Pacific (Vanuatu Islands) showed extracellular associations with bacteria on their gills that might be examples of extracellular symbiosis [13]. In addition, several morphotypes of unidentified mytilids collected from sunken wood in the Bohol Sea, the Philippines, harbored bacteria extracellularly on their gill surfaces [27].

Little is known about mytilid symbiosis for specimens collected from whale falls. *Idas washingtonia*, which was collected from whale carcasses off California at a depth of 1240 m, showed endosymbiotic relationships with thioautotrophic bacteria although a precise location for the symbiont was not included in the report [9]. *Adipicola crypta* showed intracellular and an unidentified mussel extracellular symbioses with thioautotrophic bacteria [28].

A dense aggregation of the mytilid mussel *Adipicola pacifica* (Dall, Bartsch & Rehder, 1938) and *A. crypta* (Dall, Bartsch & Rehder, 1938) was discovered at whale falls in the northwest Pacific at depths of 219–254 m in 2003 [10], [11]. *A. pacifica* covered bone surfaces exposed to seawater, while *A. crypta* was found attached only to the bones buried in sediments [11]. This presents an opportunity to compare symbiotic mussels from relatively shallow-water with mussels collected from other deeper, reducing environments.

The aims of the present study were to: (1) ascertain if whale-fall *Adipicola* species harbor symbiotic bacteria in their gills similar to other bathymodiolin mussels; (2) describe the location and phylogeny of the symbionts; and (3) consider the evolution of symbiont-harboring mussels. The symbionts were characterized using 16S ribosomal RNA (16S rRNA) gene sequences; localization was assessed by fluorescence *in situ* hybridization (FISH) experiments and electron microscopic observations. The importance of biogenic reducing environments during the evolution of symbiont-harboring invertebrates in the deep sea is discussed.

## Results

### Electron microscopic observations

**Figure 1:**
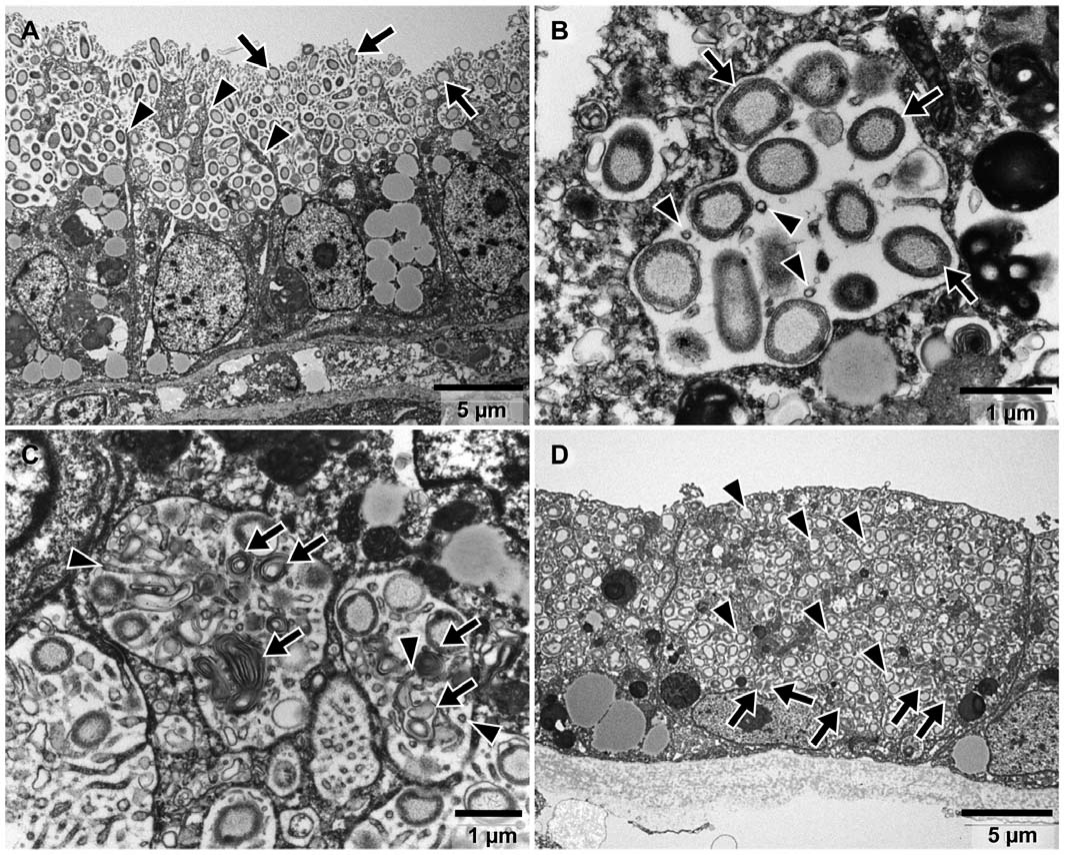
*Adipicola* mussels. Transmission electron micrographs of transverse sections of ctenidial filaments. (A)–(C) *Adipicola pacifica*. (A) Epithelial cells of the ctenidial filament. Gram-negative bacterial symbionts (arrows) are visible on the surface of the cells. Arrowheads indicate pseudopodium-like structures. (B) Bacterial symbionts (arrows) contained in vacuoles accompanied by microvilli (arrowheads). (C) Intracellular degradation of symbionts. Relics of decomposed bacteria (arrows) located in vacuoles of host cells and accompanying host microvilli (arrowheads). (D) *Adipicola crypta*. Intracellular gram-negative symbiotic bacteria within epithelial cells of the ctenidial filament. Arrowheads indicate the symbionts in vacuoles and arrows indicate digested bacteria in lysosomes.

**Figure 2:**
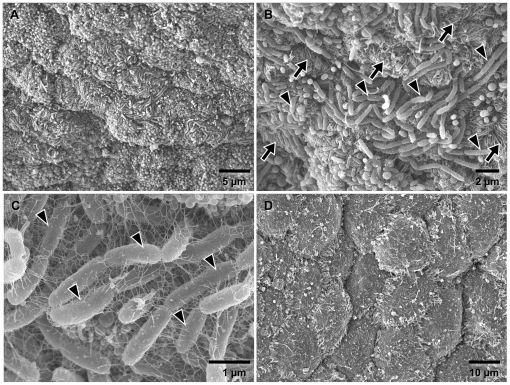
*Adipicola* mussels. Scanning electron micrographs of gill surfaces. (A)–(C) *Adipicola pacifica*. (A) & (B) Well-developed microvilli and numerous bacterial symbionts on the gill surface. Arrowheads indicate the symbionts and arrows indicate microvilli. (C) Higher magnification of the bacterial symbionts. Well-developed filamentous networks are visible. Arrowheads indicate the symbionts. (D) *Adipicola crypta*. The gill surface was flat and smooth and few bacteria are visible.

Numerous bacteria were observed in sections of ctenidial filaments of both *Adipicola* species, but the location within host species differed (Fig. 1). In *A. pacifica*, the bacteria were located on the apical surfaces of epithelial cells of the gills (Fig. 1A). Well developed “Pseudopodium-like structures (PLS)” of host cells (Fig. 1A) and a dense aggregation of microvilli were seen on cell surfaces (Figs. 1A, 2A, B) such that the bacteria were surrounded by the PLS and microvilli (Fig. 1A).

In *A. crypta* specimens, the bacteria were located in vacuoles within epithelial cells of the ctenidial filaments (Fig. 1D). The surfaces of epithelial cells were relatively smooth and there were few sparse microvilli (Figs. 1D, 2D).

In both species, the bacteria were small cocci or short rods that averaged 0.89 µm (SD = 0.15, *n* = 50) along the major axis in *A. pacifica* and averaged 0.82 µm (SD = 0.16, *n* = 50) along the major axis in *A. crypta*. Bacteria from both species showed a thin peptidoglycan layer typical of gram-negative bacteria and did not contain membranes or other distinctive structures in their cytoplasm (Fig. 1). Apart from external form (i.e., cocci or short rods), bacteria morphology was uniform. Divisional stages of the bacteria were sometimes seen (data not shown).

In *A. pacifica*, vacuole-like structures, containing microvilli and fine or partially digested bacteria, were observed in the host epithelial cells of the ctenidial filaments (Fig. 1B, C) and well-developed filamentous networks were attached to the symbionts on the cell surfaces (Fig. 2C).

In *A. crypta*, secondary lysosomes containing the intermediate stages of bacterial digestion were observed concentrated near the basal portion of the host epithelial cells (Fig. 1D).

### Molecular phylogenetic analyses of bacterial 16S rRNA sequences

Partial sequences (≈500 bp) of bacterial (16S rRNA) genes from the gill tissues of *A. pacifica* and *A. crypta* were determined.

A total of 432 clones were analyzed from seven specimens of *A. pacifica*. Two different sequences, referred to as Symbiont A and Symbiont C, appeared in the ratio of 13 to 12 ranging from 0∶1 to 1∶0 (n = 7). Four specimens harbored both types of symbionts, two harbored only Symbiont A and one harbored only Symbiont C.

A total of 274 clones were sequenced from three specimens of *A. crypta* and were homogeneous.

Nearly complete sequences of 16S rRNA genes from Symbiont A, Symbiont C and the *A. crypta* symbiont were determined using three clones from each group and were 1456 bp, 1478 bp and 1456 bp length respectively.

**Figure 3:**
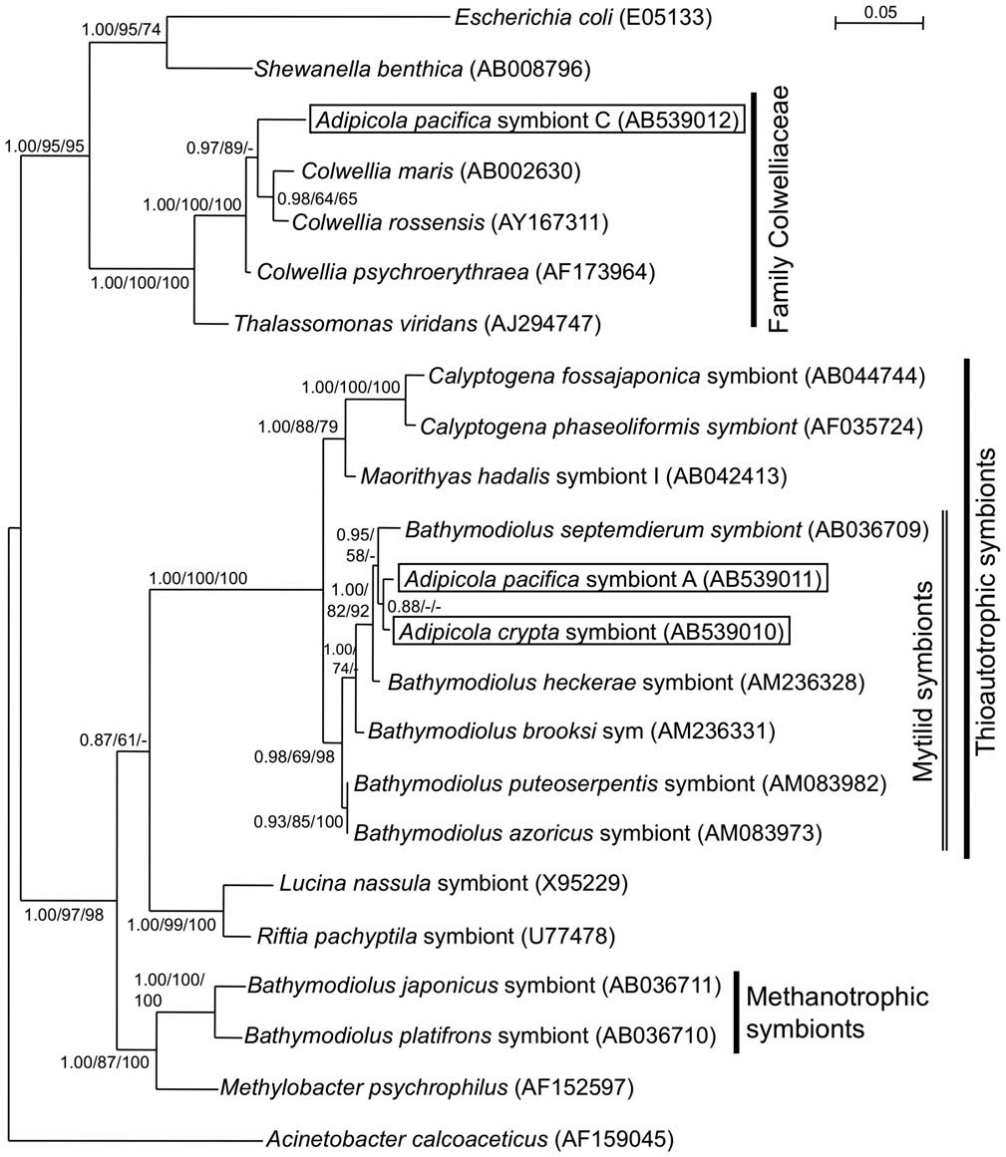
Phylogeny of bacterial symbionts from whale-fall *Adipicola* mussels based on 16S rRNA gene sequences. Bayesian (BA) tree of the γ-Proteobacteria are shown. Scale bar represents 0.05 nucleotide substitution per sequence position. A BA posterior probability greater than 0.5 and bootstrap values greater than 50% are shown for each branch, with left, center and middle values representing posterior probability in BA and bootstrap values in the maximum-likelihood (ML) and neighbor-joining (NJ) methods, respectively. Symbionts of the mussels examined in this study are highlighted. The accession numbers used for this study are shown in parentheses following the operational taxonomic unit names.

Phylogenetic analyses using Bayesian (BA), neighbor-joining (NJ) and maximum likelihood (ML) methods placed the sequences of Symbiont A, Symbiont C and the *A. crypta* symbiont within the γ subdivision of Proteobacteria containing thioautotrophic and methanotrophic symbionts associated with marine invertebrate hosts (data not shown). The three sequences from the symbionts sampled were unique to the host mussels (Fig. 3).

In all three phylogenetic analyses, Symbiont A and the *A. crypta* symbiont consistently fell into a clade with thioautotrophic symbionts of mytilids (Fig. 3). The posterior probability in BA analysis (0.98) and the bootstrap value in NJ analysis (98%) demonstrated the monophyly of this clade, although the bootstrap value in ML analysis (69%) was not high (Fig. 3).

The sequence for Symbiont C differs and formed a monophyletic clade with the free-living bacterial genus *Colwellia* (Fig. 3). The posterior probability of 1.00 in BA analysis and bootstrap values of 100% in ML and NJ analyses strongly supported the monophyly of this clade (Fig. 3).

### Fluorescence in situ hybridization (FISH)

FISH experiments were conducted on sections of gill or muscle tissues of *A. pacifica* using four different probes (SymA, SymCx, EUB338 and SymMx) and on those of *A. crypta* using three different probes (SymAc, EUB338 and SymMx).

**Figure 4:**
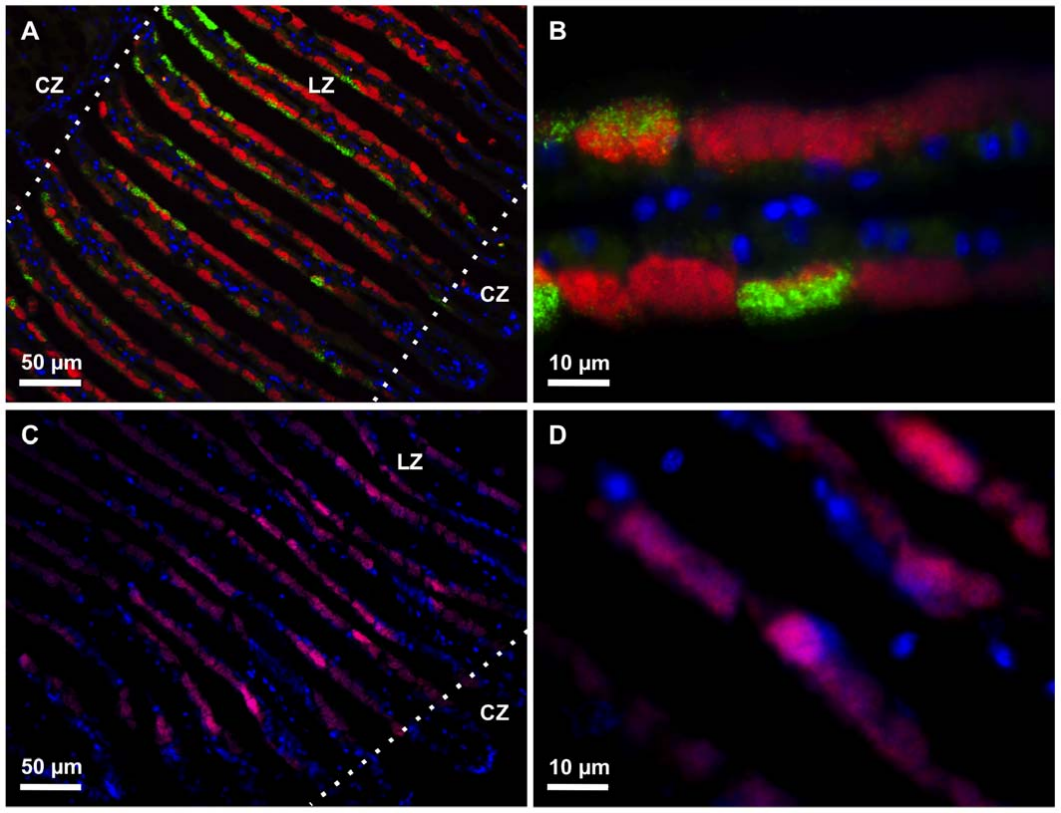
*Adipicola* mussels. Images of Fluorescence *in situ* hybridization (FISH) microscopy of bacterial symbionts in transverse sections of gill filaments of *A. pacifica* (A, B) and *A. crypta* (C, D) are shown. Hybridizations with the Symbiont A-specific probe SymA labeled with Alexa 647 (shown in red) and the Symbiont C-specific probe SymCx labeled with Alexa 555 (shown in green) are shown in A and B. Hybridizations with the *A. crypta* symbiont-specific probe SymAc labeled with Alexa 647 (shown in pink) are shown in C and D. All images are embedded sections (4-µm thickness) that were also stained with DAPI after hybridization (shown in blue). CZ: ciliated zone, LZ: lateral zone.

SymA, SymCx and EUB338 hybridized with sections of gill tissues from *A. pacifica* (Fig. 4A, B) but not with sections of muscle tissues (data not shown). The negative control probe SymMx did not hybridize with sections of any tissues (data not shown). The hybridization reactions of SymA and SymCx were localized on the apical surfaces of epithelial cells of gill tissues. The hybridization patterns of the two probes were nearly alternative, although both overlapped in some limited regions (Fig. 4A, B).

SymAc and EUB338 hybridized with sections of gill tissues from *A. crypta* (Fig. 4C, D) but not with sections of muscle tissues (data not shown). The negative control probe SymMx did not hybridize with sections of any tissues (data not shown). The hybridization reactions of SymAc were localized in the apical regions of epithelial cells of gill tissues.

### Molecular phylogenetic analyses of mussel sequences

Partial sequences of 18S ribosomal RNA (18S rRNA), cytochrome c oxidase subunit I (COI) and NADH dehydrogenase subunit 4 (ND4) gene sequences were determined using total DNA extracted from foot tissues of *A. pacifica* and *A. crypta*. Each gene from the two *Adipicola* species was aligned with homologues from 19 other mussel species. The alignable positions of the three genes from each species were combined in a total length of 2,692 bp, i.e., 520 bp of COI, 504 bp of ND4 and 1,668 bp of 18S rRNA genes.

**Figure 5:**
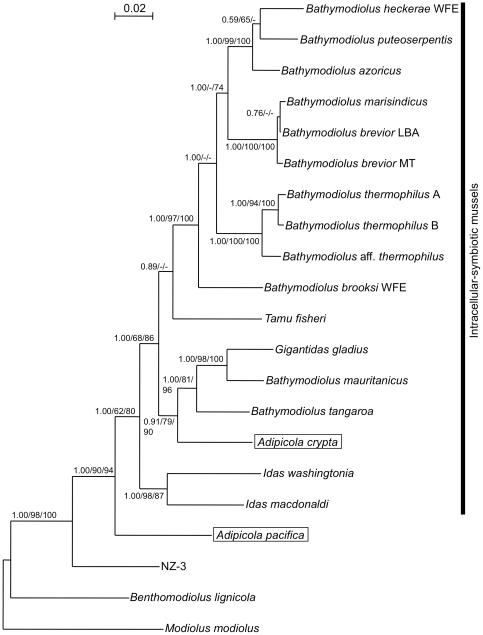
Phylogeny of whale-fall *Adipicola* mussels based on sequences of three eukaryotic genes: 18S rRNA, cytochrome c oxidase subunit I (COI) and NADH dehydrogenase subunit 4 (ND4). BA tree of mytilid mussels is shown. Scale bar represents 0.02 nucleotide substitution per sequence position. BA posterior probability greater than 0.5 and bootstrap values greater than 50% are shown for each branch, with left, center and middle values representing posterior probability in BA and bootstrap values in ML and NJ, respectively. *Adipicola* mussels examined in this study are highlighted. The accession numbers used for this study are shown in Table 2.

Three phylogenetic trees created using the BA, ML and NJ methods yielded similar (but not completely identical) topologies (Fig. 5). These three analyses showed that *A. pacifica* was a sister group of other intracellular-symbiotic mussels (Fig. 5).

The monophyly of the clade containing *A. pacifica* and intracellular-symbiotic mussels was supported by a posterior probability of 1.00 in the BA tree (Fig. 5). The three phylogenetic analyses demonstrated the monophyly of the intracellular-symbiotic mussel group, supported by the posterior probability of 1.00 in the BA tree (Fig. 5).

*A. crypta* was associated with other intracellular-symbiotic mussels and formed a monophyletic group with three other deep-sea mussels, *Gigantidas gladius*, *Bathymodiolus mauritanicus* and *Bathymodiolus tangaroa*, inhabiting hydrothermal vents or seeps in all three phylogenetic trees (Fig. 5).

### Stable isotopic analyses

**Figure 6:**
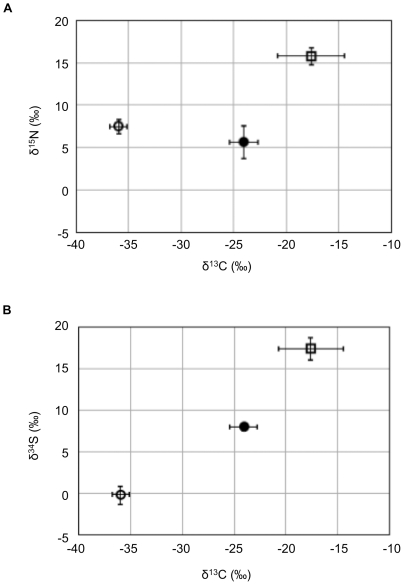
Stable isotopic compositions of soft tissues of whale-fall *Adipicola* mussels and whale tissues. (A) The δ^13^C and δ^15^N. (B) The δ^13^C and δ^34^S. Open circle: *A. crypta*, solid circle: *A. pacifica*, open square: whale tissue. Each error bar indicates standard deviation among specimens.

Stable isotopic compositions of carbon, nitrogen and sulfur obtained from the soft tissues of *A. pacifica* and *A. crypta* together with the associated whale remains and acid-volatile sulfide (AVS) in the substrate sediments were analyzed. The isotopic ratios of the whale remains showed representative values for marine animals with a high nutritional level, characterized by high δ^13^C and δ^15^N values (Fig. 6). The isotopic ratios of the *Adipicola* soft tissues were significantly lower than those of the whale tissues (Fig. 6). The stable carbon and sulfur isotopic compositions of *A. pacifica* were higher than those of *A. crypta* (Fig. 6).

## Discussion

To the best of our knowledge, this is the first report to show mixotrophic symbiosis in whale-fall mussels and also the first evidence for nutritional reliance of mussels on heterotrophic bacterial symbionts. Most *Bathymodiolus* species harbor either thioautotrophs or methanotrophs, or both in the epithelial cells of their gill filaments. One exception is *Bathymodiolus heckerae* collected from seeps in the Gulf of Mexico. This mussel species was reported to harbor four phylotypes of symbionts, a methanotroph, two phylogenetically distinct thiotrophs and a methylotroph-related phylotype not previously described from any marine invertebrate symbiosis [29]. Another exception occurs in the subfamily Bathymodiolinae. *Idas* sp. from a seep area located north of the Nile deep-sea fan (eastern Mediterranean) harbored six bacterial phylotypes, including two distinct thiotrophs, two methanotrophs and two belonging to groups not yet reported as symbionts in mytilids [30].

In the present study, *A. crypta* showed intracellular symbiosis with a single phylotype of thioautotrophic bacteria which was closely related to other thioautotrophic symbionts in deep-sea mussels from reducing environments such as hydrothermal vents and seeps (Figs. 1,2,3,4). The 16S rRNA gene sequence from *A. crypta* symbionts was identical, except for one-base insertion, to that of the previously described *A. crypta* symbionts [28]. Electron microscopic observations showed that the ctenidial filaments of our *A. crypta* specimens were relatively thick and the symbionts were located in vacuoles in ctenidial epithelial cells, which was similar to that of *A. crypta* collected from whale bones but not to the one collected on sunken wood in the previous study [27].

Molecular phylogenetic analyses showed that *A. crypta* belonged to a clade of intracellular symbiotic mussels (Fig. 5), which is consistent with the previous study [28].

Stable isotopic analyses also confirms these species rely on chemoautotrophy (Fig. 6). The isotopic ratios of *A. crypta* are similar to observations from other chemosynthesis-based thiotrophic bivalves (δ^13^C = −35±5‰, δ^34^S≤+11‰, e.g., [31]) and nearly identical to those of the whale-fall clam *Solemya pervernicosa* collected simultaneously with the *Adipicola* mussels [32]. *S. pervernicosa* also harbored thioautotrophic symbionts [32]. All of these results were consistent with thioautotrophic symbioses in deep-sea mytilids reported in previous studies [7], [28], [33], [34].

The total number of symbionts of *A. crypta* from whale bones was much greater than numbers from sunken wood [28]. Numbers of symbionts may be correlated with sulfide concentrations [28]. In samples taken off Cape Nomamisaki, Japan, *A. crypta* was attached only to the bone surfaces buried in sediments [11]. High concentrations of sulfide were reported in sediments beneath the whale carcasses [11]. This implies growth of *A. crypta* symbionts may be enhanced by high concentrations of sulfide.

In contrast symbiosis in *A. pacifica* were extracellular (Figs. 1A, B and 2A, B). Most bacteria were located on the apical surfaces of epithelial cells in lateral zones of ctenidial filaments. Some were found within vacuoles of ctenidial cells, similar to intracellular symbionts (Fig. 1B). However, those bacteria contained within host vacuoles co-occurred with microvilli found mainly on cell surfaces (Fig. 1B). This implies that gill epithelial cells phagocytosed bacteria accompanying the microvilli into cell vacuoles. Similar morphology was reported in deep-sea mussels from sunken wood sampled from waters off Vanuatu and in the Bohol Sea [27], [28]. Furthermore, bacteria in the vacuoles appeared to be digested (Fig. 1C), which implies host mussels consume bacteria by intracellular digestion, similar to bathymodiolin mussels that harbor intracellular symbionts [7], [15], [35]. In comparison with extracellular symbionts on gill tissue of sunken-wood mussels [13], [27], [28], [36], the surface structures of ctenidial epithelial cells were well developed in *A. pacifica* (Fig. 1A). Most extracellular symbiotic mussels harbor relatively few symbionts on smooth gill surfaces [13], [27], [28], [36]. The pseudopodium-like structures in *A. pacifica* increase cell surface and form “hollow” structures on the apical surfaces. Numerous symbionts were observed in the hollows (Fig. 1A). These morphological features might contribute to the phagocytosis of symbionts on a relatively large scale to that in wood-fall mussels.

The digestive system of *A. pacifica* looked similar to other bathymodiolin filter-feeders [37], [38] and we suggest this species might acquire food by filter feeding. However, the mussels were only found on nutrient-rich bone surfaces and never on exhausted bones or substrates around the whale carcasses where many suspension feeders such as *Heteralepas* barnacles, cirripeds, crinoids, cnidarians and the benthic ctenophore *Lyrocteis imperatoris* occur [11]. If the mussel primarily relies on filter feeding, it should be able to live in these environments. In addition, *A. pacifica* extended their long inhalent siphon far from the bones into the water column [10]. The biomass is quite rich on the surface of the whale bones [11] but decreases rapidly away from the surface into the water column. Efficient filter feeders should acquire more food from suspended organic particles such as bacterial mats and filaments, plankton, body wastes and secretions from the whale-fall fauna as close as possible to the bone surfaces. The implication is that *A. pacifica* does not rely on filter feeding for energy and nutrients.

From molecular phylogenetic analyses and FISH experiments, there are two phylotypes of bacteria on the *A. pacifica* gill (Figs. 3, 4A, B). The first, Symbiont A, was clearly included within a clade of thioautotrophic symbionts from the gill tissues of bivalves such as vesicomyid clams, bathymodiolin mussels and a thyasirid clam (Fig. 3). The expression of the sulfur oxidation B gene (*SoxB*) was detected in the gills but not in the foot of *A. pacifica* (Fujiwara et al., unpublished data). All mytilids from sunken wood and whale falls examined to date harbored thioautotrophic symbionts [27], [28]. Taken together, the results strongly support the symbiotic relationship between thioautotrophic Symbiont A and *A. pacifica*.

In contrast Symbiont C phylotype in *A. pacifica*, was not closely related to any other known symbionts (Fig. 3). The closest relatives were the free-living heterotrophic bacterial genus *Colwellia*, which is a psychrophilic, gram-negative bacterium that can be found in continually cold marine environments including the deep sea [39]–[41]. It is possible that Symbiont C is a contaminant from these environments but it has never been reported from bone surfaces or sediments underneath the whale bones using molecular techniques (data not shown). In addition, the FISH results clearly show that both Symbiont A and Symbiont C were associated with the ctenidial filaments of *A. pacifica* (Fig. 4A, B). It is unlikely that a mass of Symbiont C was entangled on the gill surfaces from the environments where no Symbiont C was recorded. We have no physiological information on Symbiont C but the genus *Colwellia* are believed to be heterotrophs. It is not clear how Symbiont C derives energy, but whale bones contain large amounts of organic materials such as lipids and proteins and these may provide nutrients. Heterotrophic symbionts in bone-eating *Osedax* polychaetes were reported to utilize organic substrates in whale bones for energy although details of nutritional processes are unknown [42].

Neither symbiont within *A. pacifica* showed specific distribution patterns within the gill tissues, unlike two symbionts of the hadal thyasirid clam *Maorithyas hadalis* that showed spatial partitioning in its gills [43]. Intracellular digestion of symbionts was observed throughout the gills (Fig. 1) implying both symbionts were digested non-selectively and incorporated into their hosts. This is consistent with the stable isotopic results (Fig. 6).

The δ^13^C and δ^34^S values in *A. pacifica* were intermediate between those for whale tissues and *A. crypta* (Fig. 6). The relatively lower δ^34^S values of *A. pacifica* strongly indicated its incomplete reliance on the photosynthesis-based nutrition that shows relatively uniform δ^34^S values close to dissolved sulfate and sulfur in seawater (δ^34^S = +21‰) [44].

The δ^13^C values of *A. pacifica* were similar to coastal species that rely on land-derived detritus [44]. The δ^13^C value of terrigenous organic matter is about −25‰ [44]. However, a total amount of such detritus was very limited at the off Nomamisaki site (data not shown). The δ^13^C value of whale remains is about −18‰ and the value for thioautotrophic production is about −35±5‰ [44]. Therefore, it is possible that *A. pacifica* utilized both carbon sources.

The δ^15^N values of *A. pacifica* and *A. crypta* were similar (δ^15^N = ca.+6–8‰). It is not easy to identify nitrogen sources as several candidates exist [45]. The δ^15^N values of symbiont-harboring invertebrates vary even within species [31]. Nitrogen isotopic analyses of each amino acid from the mussels and the bones and of ammonium in the sediments and the bones may clarify the precise nitrogen sources [46]. The results from the present study imply that *A. pacifica* rely on both thioautotrophic Symbiont A and heterotrophic Symbiont C.

**Figure 7:**
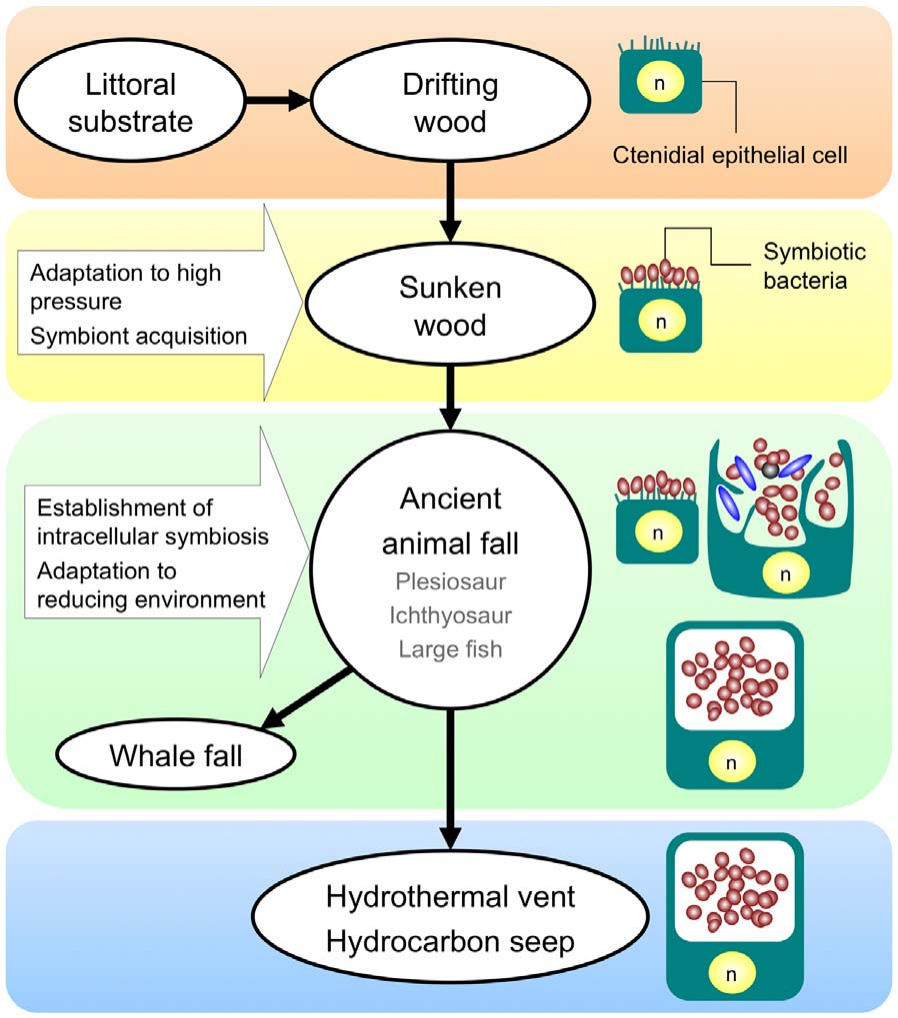
Hypothetical schemes for the evolution of symbiont-harboring mytilids. Mussel habitats and representative symbiotic forms in mussels from each habitat are shown. Open ellipse: mussel habitat, solid arrow: emigration of mussel, n: nucleus.

The diverse symbiotic forms observed in the family Mytilidae seemed to be correlated to the mussel habitats, which provide some idea for the evolution of deep-sea mussels and symbiosis. Symbiont-harboring bathymodiolin mussels are thought to have been derived from their asymbiotic shallow-water relatives by way of sunken woody plant materials [18] (Fig. 7). Coastal mussel species are known to attach drifting wood [47]–[49] that would sink during decomposition. Live specimens of shallow-water mussel *Mytilus galloprovincialis* were found on sunken wood at 110 m (Haga, pers. comm.). The supply of organic material in deep sea is limited. Sunken wood can produce reducing environments that are able to nourish bacteria including heterotrophs that use wood directly and thioautotrophs that use reducing sulfur compounds released from the wood as electron acceptors for chemosynthesis. It is conceivable that asymbiotic ancestors of symbiont-harboring mussels might have adapted to moderate reducing environments as filter feeders and the symbiotic relationship between mussels and thioautotrophic bacteria was established afterwards.

Most sunken-wood mytilids show extracellular symbioses, which is thought to be an earlier form of symbiosis [23]. The ctenidial epithelial cells of sunken-wood mussels are reported to be smaller than those of bathymodiolin mussels from hydrothermal vents and seeps and the total number of symbionts was also markedly fewer [4], [7], [13], [28], [36]. This implies that sunken-wood mussels do not wholly rely on their symbionts for nutrition. Molecular phylogenetic analyses also support an early divergence of sunken-wood mytilids within symbiont-harboring mussels [12], [18], [28], [50].

A wide variety of symbiotic forms appear in mussels from whale-fall environments, which may reflect a wide range of redox states around whale carcasses (Fig. 7). Sulfide concentrations were higher in deeper sediments than in shallower ones beneath the carcasses and undetectable in seawater surrounding the bone surfaces [11]. In Japanese waters, three other mussel species have been collected from whale bone surfaces, which we presume have low sulfide concentrations. All these species show extracellular symbioses similar to those of *A. pacifica* (Fujiwara et al., unpublished data).

In contrast, most endosymbiotic mussels such as *Bathymodiolus* species from hydrothermal vents and seeps and *A. crypta* from bone surfaces buried in sediments inhabit highly reducing environments. An intracellular symbiosis may be a necessary precondition to enable settlement of mytilids in sulfide-rich environments, although other episymbiotic taxa such as thyasirid clams and the symbiont-harboring ciliate *Zoothamnium niveum* are able to inhabit such reducing environments [51], [52]. Whale falls provide a range of reducing environments that might provide an opportunity for symbiont-harboring mussels to adapt to different redox conditions. In turn, symbioses could evolve from extra- to intracellular types under selection pressure for the most stable efficient nutritional intake.

However according to the fossil records, larger whales evolved after *Bathymodiolus* species at seeps. Before the existence of whales in the Mesozoic, it is speculated that large marine vertebrates such as ichthyosaurs, plesiosaurs, and large fishes harbored chemosynthesis-based biological assemblages [53]–[55]. In fact, the first Mesozoic occurrences of chemosynthesis-based communities developed on large marine plesiosaurid carcasses have been reported although no symbiont-harboring invertebrates including mussels have not yet found [56].

Taken together, the results imply that mussel species living in present day vents and seeps have been derived from sunken-wood relatives by way of ancient animal falls. Whale-fall environments and other modern biogenic reducing environments might have served as refugia for ancient lineages of mussels showing less-integrated symbiotic forms (Fig. 7). The possibility that the extracellular symbiotic pioneers first appeared at seeps or vents and that a more integrated progeny showing intracellular symbioses took the place of its ancestors cannot be excluded, although there has been no record of extracellular symbiotic mussels from either vents or seeps.

## Materials and Methods

### Specimen collection

*A. pacifica* and *A. crypta* specimens were collected off Cape Nomamisaki, Japan, during R/V *Natsushima*/ROV *Hyper-Dolphin* cruises NT03-08, NT04-08 and NT05-12 in 2003, 2004 and 2005, respectively. Upon recovery, the mussels were immediately transferred to fresh, chilled (12°C) seawater.

### Treatment for electron microscopic observation

Small pieces of gill tissue of *A. pacifica* (n = 20) and *A. crypta* (n = 5) were fixed with 2.5% glutaraldehyde in filtered seawater for 24 hours and preserved in filtered seawater with 10 mM sodium azide at 4°C. Samples were then washed in filtered seawater and fixed with 2% osmium tetroxide in filtered seawater for 2 hours at 4°C. For scanning microscopic observations, gill tissues were rinsed with distilled water and incubated with 1% aqueous tannic acid (pH 6.8) for 1 hour for conductive staining. These samples were again washed with distilled water and treated with 1% aqueous osmium tetroxide for 1 hour. The gill tissues were dehydrated in a graded ethanol series and critical point-dried using a JCPD-5 critical point dryer (JEOL, Akishima, Japan). The samples were coated with osmium using a POC-3 osmium plasma coater (MEIWAFOSIS Co., Osaka, Japan). The coated tissues were then observed using a JSM-6700F field-emission scanning electron microscope (JEOL) at an acceleration voltage of 5 kV.

For transmission electron microscopic observations, the gill tissues were rinsed with distilled water and stained *en bloc* with 1% aqueous uranyl acetate for 2 hours at 4°C. Those samples were rinsed with distilled water, dehydrated in a graded ethanol series and embedded in Epon 812 resin (TAAB, Aldermaston, UK). Ultrathin sections were prepared using a Reichert Ultracut S ultra microtome (Leica, Vienna, Austria). The gill sections were stained with aqueous uranyl acetate and Sato's lead and then observed with a JEM-1210 transmission electron microscope (JEOL) at an acceleration voltage of 100 kV.

### DNA preparation

DNA was extracted from the gill tissues and feet of *A. pacifica* and *A. crypta*. To reduce surface contaminants, each tissue sample was thoroughly washed in autoclaved and filtered (0.22 µm) seawater. DNA extraction from tissue samples was conducted separately using a DNeasy Tissue Kit (Qiagen Japan, Tokyo, Japan).

### Polymerase chain reaction (PCR) amplification, cloning and sequencing

The *Adipicola* mussels were examined for three genes: COI, ND4 and 18S rRNA. The corresponding symbionts were examined for the 16S rRNA gene. PCR amplification was conducted using an Ex Taq PCR kit (TaKaRa, Kyoto, Japan). Two oligonucleotide primers (0.2 µM each) and <1 µg of DNA template were added to the reaction mixtures. Thermal cycling was: denaturing at 96°C for 20 seconds, annealing at 55°C for 45 seconds and extension at 72°C for 2 minutes for a total of 35 cycles. The oligonucleotide primer sequences used for the PCR amplifications are shown in Table 1. The molecular sizes of the PCR products were confirmed with 1.2% Agarose S (Nippon Gene, Toyama, Japan) gel electrophoresis. The PCR products were purified using the Wizard SV Gel and PCR Clean-Up System (Promega, Madison, WI, USA). For bacterial genes, the PCR amplicons were cloned into the pCR-TOPO vectors using a TOPO TA cloning kit (Invitrogen, San Diego, CA, USA). The DNA constructs were transferred into *Escherichia coli* TOP10 cells (Invitrogen). The DNA sequencing reaction of the bacterial 16S rRNA gene clones and the amplified eukaryotic COI, ND4 and 18S rRNA genes was performed using a BigDye Terminator v3.1 Cycle Sequencing Kit (Applied Biosystems, Foster City, CA, USA). Specific primers for each gene (Table 1) were used in sequencing reactions according to the manufacturer's recommended procedure. Sequencing was performed using an ABI PRISM 3100 genetic analyzer (Applied Biosystems). The sequences reported here have been deposited in the DDBJ database under accession numbers AB539004, AB539005, AB539006, AB539007, AB539008, AB539009, AB539010, AB539011 and AB539012.

**Table 1:** Oligonucleotide primers and probes used for PCR amplification, sequencing and fluorescence *in situ* hybridization (FISH).

Target gene		Primer/probe	Sequence (5′→3′)	Orientation	Use
Bacterial	16S rRNA	27F	AGAGTTTGATCCTGGCTCAG	Forward	PCR/Sequencing
		1492R	GGTTACCTTGTTACGACTT	Reverse	PCR/Sequencing
		350F	TACGGGAGGCAGCAG	Forward	Sequencing
		786F	GATTAGATACCCTGGTAG	Forward	Sequencing
		1100F	GCAACGAGCGCAACCC	Forward	Sequencing
		1224F	TACACACGTGCTACAATG	Forward	Sequencing
		519R	GTATTACCGCGGCTGCTG	Reverse	Sequencing
		785R	CTACCAGGGTATCTAATCC	Reverse	Sequencing
		1225R	CCATTGTAGCACGTGTGT	Reverse	Sequencing
		SymA	TCGCCACTAAGAGGTAAATCCC	Reverse	FISH
		SymCx	TTAGCTGCGCCACTCACGTCTC	Reverse	FISH
		SymAc	TCGCCACTAAGAGGTAAATCCTC	Reverse	FISH
		BAC338	ACTGCTGCCTCCCGTAGGAGTCT	Reverse	FISH
		SymMx	CCGCCACTAAACCTGTATATA	Reverse	FISH
Eukaryotic	18S rRNA	1N	TCCTGCCAGTAGTCATATGC	Forward	PCR/Sequencing
		2N	TGATCCTTCT/CGCAGGTTCAC	Reverse	PCR/Sequencing
		555F	AGTCTGGTGCCAGCAGCCGC	Forward	Sequencing
		555R	GCGGCTGCTGGCACCAGACT	Reverse	Sequencing
		1269R	AAGAACGGCCATGCACCAC	Reverse	Sequencing
		1269F	GTGGTGCATGGCCGTTCTT	Forward	Sequencing
	COI	LCO1490	GGTCAACAAATCATAAAGATATTGG	Forward	PCR/Sequencing
		HCO2198	TAAACTTCAGGGTGACCAAAAAATCA	Reverse	PCR/Sequencing
	ND4	Arg BL	CAAGACCCTTGATTTCGGCTCA	Forward	PCR/Sequencing
		NAP 2H	TGGAGCTTCTACGTGRGCTTT	Reverse	PCR/Sequencing

**Table 2:** Operational taxonomic units (OTUs) used for phylogenetic analysis of host mussels and bacterial symbionts.

Mytilid OTU	Accession number			Habitat
	COI	ND4	18S rRNA	
*Adipicola crypta*	**AB539004**	**AB539006**	**AB539008**	Whale fall
*Adipicola pacifica*	**AB539005**	**AB539007**	**AB539009**	Whale fall
*Bathymodiolus aff. thermophilus*	AF456317	AY649809	AY649823	Vent
*Bathymodiolus azoricus*	AY649795	AF128534	AY649822	Vent
*Bathymodiolus brevior* LBA	AY275544	AY046277	AY649827	Vent
*Bathymodiolus brevior* MT	AY649799	AY649806	AY649824	Vent
*Bathymodiolus brooksi* WFE	AY649798	AY649805	AY649825	Seep
*Bathymodiolus heckerae* WFE	AY649794	AY130246	AF221639	Seep
*Bathymodiolus marisindicus*	AY275543	AY046279	AY649818	Vent
*Bathymodiolus mauritanicus*	AY649801	AY649810	AY649828	Seep
*Bathymodiolus puteoserpentis*	AY649796	AF128533	AF221640	Vent
*Bathymodiolus tangaroa*	AY608439	AY649811	AY649820	Seep
*Bathymodiolus thermophilus* A	AF456285	AY649807	AF221638	Vent
*Bathymodiolus thermophilus* B	AF456303	AY649808	AY649829	Vent
*Benthomodiolus lignicola*	AY275545	AY649817	AF221648	Sunken wood
*Gigantidas gladius*	AY649802	AY649813	AY649821	Vent
*Idas macdonaldi*	AY649804	AY649816	AF221647	Seep
*Idas washingtonia*	AY275546	AY649815	AF221645	Whale fall & sunken wood
*Modiolus modiolus*	U56848	EF526453	EF526454	Littoral
NZ3	AY608440	AY649812	AY649819	Vent
*Tamu fisheri*	AY649803	AY649814	AF221642	Seep

Associated DDBJ accession numbers original to this study are shown in boldface type.

### Phylogenetic analysis

Partial sequences of the bacterial 16S rRNA genes and eukaryotic COI, ND4 and 18S rRNA genes were analyzed using the gapped-BLAST search algorithm [57], [58] to estimate the degree of similarity to other relative sequences. Sequences of approximately 1,500 bp (16S rDNA), 530 bp (COI), 530 bp (ND4) and 1,700 bp (18S rDNA) were used for the similarity analyses. The non-redundant nucleotide sequence database of the DNA Data Bank of Japan was used for similarity analyses.

Sequences were aligned using Clustal X [59], followed by manual editing of the resulting alignments. Phylogenetic analyses were restricted to nucleotide positions that were unambiguously alignable in all sequences. The alignments (23 taxa/1,358 bp for bacterial 16S rRNA genes and 21 taxa/2,692 bp for eukaryotic COI+ND4+18S rRNA genes) are available on request from the corresponding author.

BA statistical analyses were conducted with MrBayes software ver. 3.1.2 [60]. The GTR+I+Γ evolutionary model was chosen for analysis of the bacterial 16S rDNA dataset using MrModeltest software ver. 2.2 [61]. Partitioned BA inference phylogenetic analyses were performed for the combinations of eukaryotic COI, ND4 and 18S rDNA with MrBayes software. Three partitions were set (COI, ND4 and 18S rDNA). The GTR+I+Γmodel was used in the analysis for the COI and ND4 datasets and the GTR+I model for the 18S rDNA dataset. The analyses were run for one million generations for the bacterial dataset and 0.5 million generations for the eukaryotic dataset, sampled every 100 generations. BA posterior probability was then calculated from the sample points after the Markov Chain Monte Carlo algorithm began to converge. ML analyses were performed using PhyML software [62] with an input tree generated by BIONJ with general time-reversible models [63] of nucleotide substitution incorporating invariable sites and a discrete gamma distribution (eight categories) (GTR+I+Γmodel). Model parameters were estimated from the dataset. The ML bootstrap analyses (500 replicates) were constructed as in the model and settings described in the preceding text. Calculation of the distance matrix and NJ analysis was accomplished using the Clustal X software package [59].

### Fluorescence in situ hybridization (FISH)

Two ribosomal RNA-targeted oligonucleotide probes, SymA and SymCx, were designed for the detection of the two types of bacteria referred to as Symbiont A and Symbiont C, respectively (Table 1). These bacteria were potentially predominant in the gill tissue of *A. pacifica*. A single rRNA-targeted oligonucleotide probe (SymAc) was designed for the detection of bacterial 16S rRNA of potential *A. crypta* symbionts in its gill (Table 1). EUB338 [64] was also used to label members of the domain Bacteria as a positive control. The sequences of SymA, SymCx and SymAc probes were analyzed using the gapped-BLAST search algorithm [57], [58] to examine whether any other sequences had similarity to these probe sequences. Although the SymA, SymCx and SymAc probes matched with many sequences deposited in database, none of the matched sequences were found in the DNA clone library established from the DNA extracts from the host mussels and environmental samples including bones.

FISH experiments were performed on transverse sections of gill tissues from three *A. pacifica* and three *A. crypta* specimens. Gill tissues were fixed in 3.7% formaldehyde in filtered artificial seawater (FAS) (4°C, 36 hours) and stored at 4°C in 70% ethanol. Tissues were embedded in O.C.T. compound (Sakura Finetek Japan, Tokyo, Japan) and 4 µm-thick sections were cut using an HM550 cryostat (Microm, Walldorf, Germany) and collected on MAS (Matsunami Adhesive Silane)-coated glass slides (Matsunami Glass Industry, Osaka, Japan). The compound was removed by rinsing three times with FAS.

Hybridization was conducted at 46°C for 2 hours in a solution containing 20 mM Tris-HCl (pH 7.4), 0.9 M NaCl, 0.1% sodium dodecyl sulfate, 25% (vol/vol) formamide and 10 µM of each probe. After hybridization, each slide was washed at 48°C for 15 min in a solution lacking the probe and formamide at the same stringency, adjusted by NaCl concentration [65]. The sections were subsequently stained with 0.4 µg/ml 4,6-diamidino-2-phenylindole (DAPI) and mounted with SlowFade Gold Antifade Reagent (Invitrogen). The slides were examined using an Eclipse E600 microscope (Nikon, Tokyo, Japan). A negative control SymMx probe was used for testing unspecific labeling. Another hybridization experiment was performed on muscle tissue from each mussel species as a negative control using the same probes described above.

### Stable isotopic analysis

For the isotope analyses, *A. crypta*, *A. pacifica* and whale remain samples obtained from whale no. 7 were used. On recovery, samples were immediately frozen at −80°C. Ten individual samples were thawed and dissected and soft tissues were divided into: gill, mantle and remaining organs. Samples were divided into two groups (five individuals each) and the each organ from each group mixed and homogenized for carbon and nitrogen analyses. For sulfur analyses, most soft tissues were used because a relatively large amount of samples were required. The whale bone sample surfaces were treated with a mixture of chloroform and methanol (3∶1 by volume) and pulverized using a propeller mill.

Pretreatment for the removal of excess seawater sulfate contained within the dissected soft tissues of the mussels was performed by reported method [66]. The soft tissues and pulverized whale bones were repeatedly dialyzed in cellulose bags at 5°C using distilled water. The samples were then freeze-dried and pulverized. A portion of the dried samples was then used for carbon and nitrogen isotopic measurement. For measurement of sulfur isotopic compositions, samples were combusted using an 1108 Oxygen Bomb (Parr Instrument Company, Moline, IL, USA) filled with oxygen under pressure of 30 kg/cm^2^ and a few milliliters of distilled water. After combustion, organic sulfur was completely converted into sulfate ions dissolved in the water. The sulfate-sulfur was then recovered by precipitation as BaSO_4_.

Carbon and nitrogen isotopic compositions were measured using a NA2500 continuous flow-isotope ratio mass spectrometer, Conflo III and Delta Plus (Thermo Fisher Scientific, Waltham, MA, USA) and are reported in the conventional δ^13^C and δ^15^N notation relative to Vienna Pee Dee Belemnite and atmospheric nitrogen, respectively. The overall reproducibility of the carbon and nitrogen isotopic analyses was ±0.1 and ±0.2‰, respectively. Sulfur isotopic compositions were measured using a SIRA 10 dual inlet stable isotope ratio mass spectrometer (VG Isogas, Cheshire, UK). BaSO_4_ was converted into SO_2_ gas *in vacuo* via its pyrolysis with a V_2_O_5_–SiO_2_ mixture, following the method described by Yanagisawa and Sakai [67]. The sulfur isotopic compositions are reported in the normal δ^34^S notation relative to Vienna Canyon Diablo troilite. The overall reproducibility of sulfur isotopic analysis, which was calculated by repeated measurement of a BaSO_4_ working standard, was ±0.1‰.
